# Overweight and General and Abdominal Obesity in a Representative Sample of Spanish Adults: Findings from the ANIBES Study

**DOI:** 10.1155/2016/8341487

**Published:** 2016-06-12

**Authors:** Ana M. López-Sobaler, Aránzazu Aparicio, Javier Aranceta-Bartrina, Ángel Gil, Marcela González-Gross, Lluis Serra-Majem, Gregorio Varela-Moreiras, Rosa M. Ortega

**Affiliations:** ^1^VALORNUT Research Group, Department of Nutrition, Faculty of Pharmacy, Complutense University of Madrid, Plaza Ramón y Cajal s/n, 28040 Madrid, Spain; ^2^Department of Preventive Medicine and Public Health, University of Navarra, C/Irunlarrea 1, 31008 Pamplona, Spain; ^3^CIBER: CB12/03/30038 Fisiopatología de la Obesidad y la Nutrición, CIBERobn, Instituto de Salud Carlos III (ISCIII), 28029 Madrid, Spain; ^4^Department of Biochemistry and Molecular Biology II, Institute of Nutrition and Food Sciences, University of Granada, Campus de la Salud, Avenida del Conocimiento, Armilla, 18100 Granada, Spain; ^5^ImFINE Research Group, Department of Health and Human Performance, Technical University of Madrid, C/Martín Fierro7, 28040 Madrid, Spain; ^6^Research Institute of Biomedical and Health Sciences & Medical School, University of Las Palmas de Gran Canaria, Edificio Departamental y de Investigación, Planta Baja Paseo Blas Cabrera Felipe “Físico” s/n, 35016 Las Palmas de Gran Canaria, Las Palmas, Spain; ^7^Department of Pharmaceutical and Health Sciences, Faculty of Pharmacy, CEU San Pablo University, Urbanización Montepríncipe, Carretera Boadilla km 5.3, Boadilla del Monte, 28668 Madrid, Spain; ^8^Spanish Nutrition Foundation (FEN), C/General Álvarez de Castro 20, 1^a^ pta, 28010 Madrid, Spain

## Abstract

*Objective*. To analyze the anthropometric parameters from a representative sample of Spanish adults participating in ANIBES study and the prevalence of general and abdominal obesity.* Methods*. This cross-sectional study focused on 1655 adults aged 18–64 years. Weight, height, and waist circumference (WC) were evaluated, and body mass index (BMI) and waist to height ratio (WHtR) were calculated. A composite index combining BMI and WHtR was designed to establish five groups with different anthropometric status.* Results*. The prevalence of overweight (OW) was 35.8% and that of obesity was 19.9%. Obesity (OB) was higher among men (OR 1.725, 1.415–2.104; *p* = 0.000) and each year of age increased the risk of obesity (OR 1.054, 1.045–1.064; *p* = 0.000). The prevalence of abdominal obesity (WHtR ≥ 0.5) was 58.4%. Only 36.1% of the population had an optimal anthropometric situation (BMI < 25 kg/m^2^, WHtR < 0.5), whereas 50.1% had weight excess and high WHtR (BMI ≥ 25 kg/m^2^, WHtR ≥ 0.5).* Conclusions*. More than half of Spanish population has weight excess and cardiometabolic risk. The results of this study provide an understanding of the current anthropometric situation in the Spanish population, as a first step toward planning interventions and assessing their effectiveness in the future.

## 1. Introduction

Obesity is a major global health problem, with 500 million obese individuals worldwide [1, 2]. It has been found that the condition of overweight and/or obesity affects more than 50% of the adult population in Spain and almost 30% of children and adolescents [3–5].

Obesity is a significant health concern because it predisposes individuals to several comorbidities, including hypertension, dyslipidemia, coronary heart disease, type 2 diabetes, stroke, cancer, and osteoarthritis [6–12], and a shortened life expectancy while impairing quality of life [13, 14]. Also, the economic cost of treating obesity-related comorbidities is very high [15, 16]. Therefore, obesity is emerging as one of the most significant health concerns of the 21st century [15].

In Spain, the National Health Survey [3] provides data on overweight and obesity every 3 years. However, the survey relies on self-reported height and weight measurements, which reduces the accuracy of the final results. Recent studies conducted on representative samples of the population have focused primarily on children [17, 18]; studies on representative samples of Spanish adults are not as recent [4, 5, 19–21].

Knowledge of the current anthropometric status of a population is important to assess its evolution and the results of any interventions [3, 4, 18]. As part of the ANIBES study (Anthropometry, Intake, and Energy Balance in Spain), the aim of this paper is to analyze the anthropometric data and to establish the prevalence of general and abdominal obesity in a representative sample of Spanish adults.

## 2. Material and Methods

### 2.1. Study Design and Sampling Procedure

The design, protocol, and methodology of the ANIBES study have been described in detail elsewhere [22–24]. ANIBES was designed to carry out an accurate updating of food and beverage intake, dietary habits and behavior, and anthropometric data of the Spanish population (9–75 years old, *n* = 2009), as well as energy expenditure and physical activity patterns.

Briefly, the sample for the ANIBES study was designed based on 2012 census data published by the INE (Instituto Nacional de Estadística/Spanish Bureau of Statistics). The total sample size was calculated based on a 0.05 probability of Type I error (rejecting a null hypothesis when it is true) and 0.1 probability of Type II error (accepting a null hypothesis when it is wrong) in the main outcome of the study (energy intake). For the total sampling, the following variables were taken into account: age, sex, geographical distribution (Northeast, Levant, South, West, North-Central, Barcelona, Madrid, and Balearic and Canary Islands), and locality size (2,000–30,000 inhabitants, rural population; 30,000–200,000 inhabitants, semiurban population; and over 200,000 inhabitants, urban population). In order to ensure the representativeness of the sample, 128 sampling points were used. No previous prerecruitment was considered in order to minimize the risk of bias in responses [25]. The present paper is focused on an adult population (excluded elderly) (*n* = 1655), considering sex (779 men/858 women) and age (883 subjects between 18 and 40 years and 772 between 41 and 64 years) [22–24].

Several exclusion criteria were applied: those individuals living in an institutional setting (e.g., colleges, nursing homes, hospitals, and others); individuals following a therapeutic diet owing to recent surgery or taking any medical prescription; potential participants with a transitory illness (i.e., flu, gastroenteritis, and chicken pox) at the time of the fieldwork; and individuals employed in areas related to consumer science, marketing, or the media [22–24].

Fieldwork for the ANIBES study took place from mid-September 2013 to mid-November 2013 (3 months). The final protocol was approved by the Ethical Committee for Clinical Research of the Region of Madrid in Spain [22–24]. Written informed consent was obtained from all subjects.

### 2.2. Anthropometric Data

Weight, height, and waist circumference were measured by trained interviewers following standardized procedures [26]. Weight was measured once with a Seca® model 804 weighing scale (Medizinische Messsysteme und Waagen seit 1840, Hamburg, Germany; range 0.1–150 kg, precision 100 g). Height was assessed in triplicate using a Seca® model 206 Stadiometer (Medizinische Messsysteme und Waagen seit 1840, Hamburg, Germany; range 70–205 cm, precision 1 mm). Waist circumference was measured in triplicate using a Seca® 201 tape measure (Seca, Hamburg, Germany; range 0–150 cm, precision 1 mm).

General obesity was assessed using body mass index (BMI) and abdominal obesity by both waist circumference (WC) and waist to height ratio (WHtR). BMI was calculated as weight (kg)/height (m)^2^. Participants were classified into the following categories: underweight (BMI < 18.5 kg/m^2^), normal weight (BMI 18.5–24.9 kg/m^2^), overweight (BMI 25–29.9 kg/m^2^), and obesity (BMI ≥ 30 kg/m^2^), based on World Health Organization International Classifications [6]. Overweight and obesity were combined into one category called “weight excess” (BMI ≥ 25 kg/m^2^) for subsequent analysis. High WC (abdominal obesity) was defined as >88 cm for women and >102 cm for men [27, 28]. WHtR was calculated as WC (cm)/height (cm). Respondents were classified into two categories: those with no abdominal obesity (WHtR < 0.5) and those with abdominal obesity with metabolic risk (WHtR ≥ 0.5) [28–31].

In addition, we defined a composite index combining BMI and WHtR groups in a five-category variable, where level 1 represents the best anthropometric situation and level 5 the worst. The five groups of this composite index are

 
*Level 1*: underweight or normal weight and WHtR < 0.5 (*n* = 597),
 
*Level 2*: overweight or obesity and WHtR < 0.5 (*n* = 92),
 
*Level 3*: underweight or normal weight and WHtR ≥ 0.5 (*n* = 137),
 
*Level 4*: overweight and WHtR ≥ 0.5 (*n* = 503),
 
*Level 5*: obesity and WHtR ≥ 0.5 (*n* = 326).

### 2.3. Statistical Analysis

Data are presented as means, standard deviation, median, percentiles, and percentages. Analyses were performed using IBM SPSS version 22.0 (IBM Corp., Armonk, NY, USA). The Kolmogorov-Smirnoff test was used to test if the variables followed a normal distribution, to decide between parametric or nonparametric analysis. Differences by age and between sexes were performed using the Mann-Whitney test. When comparing proportions, the *Z*-test was used. A Kruskal-Wallis test was used to test differences by BMI, WC, WHtR, and composite index, and the Dunn-Bonferroni* post hoc* method was applied. Spearman correlation was used for ordinal variables and Pearson correlation for ratio variables.

The influence of sex and age on risk classification of general and abdominal obesity was analyzed by logistic regression analysis to calculate odds ratios (OR). The dependent variables were BMI, WC, WHtR, and composite index groups. The reference groups were normal weight (BMI 18.5–24.9 kg/m^2^), no abdominal obesity regarding WC (WC below specific sex cutoff point), no abdominal obesity regarding WHtR (WHtR < 0.5), and level 1 composite index (BMI < 25 kg/m^2^ and WHtR < 0.5). The 95% confidence intervals (CI) were calculated, and Wald test was used for comparison of the OR. The level of significance was set at *p* < 0.05.

## 3. Results

The sample included 1655 adults (51.8% women).  Table 1 shows the anthropometric data for the entire sample and by sex and age group (18–40 years and 41–64 years). The prevalence of underweight was 1.8%.

The prevalence of overweight and obesity was 35.8% and 19.9%, respectively. This means that 55.7% of the whole sample (63.1% of men and 48.7% of women) had weight excess (BMI ≥ 25 kg/m^2^). Weight, height, BMI, and prevalence of overweight and obesity were higher among men and those in the older age group (41–64 years) (*p* < 0.05) (Table 1).

Significant positive correlations were found between age and weight, BMI, WC, and WHtR in the entire sample (*r* = 0.193, *r* = 0.342, *r* = 0.399, and *r* = 0.479, resp.; *p* = 0.000 in all cases) and for both sexes (data not shown). In all groups, the strongest correlations were found for WHtR.

Men are more likely to have BMI ≥ 25 kg/m^2^ (OR 1.725, 1.415–2.104; *p* = 0.000) and to be obese (OR 1.746, 1.341–2.273; *p* = 0.000). Each year of age also increased the likelihood of having BMI ≥ 25 kg/m^2^ (OR 1.054, 1.045–1.064; *p* = 0.000) and obesity (OR 1.073, 1.060–1.086; *p* = 0.000).

**Figure 1:**
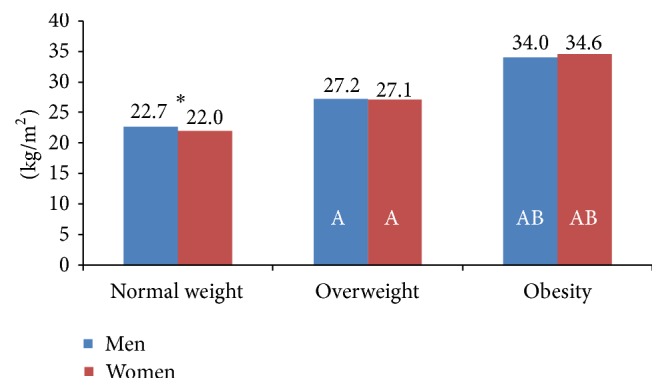
BMI in Spanish adults according to BMI classification. ^*∗*^Significant differences between men and women in the same BMI group (Mann-Whitney *U* test). Significant differences in the same sex group, A: regarding normal weight and B: regarding overweight (Kruskal-Wallis test and Dunn-Bonferroni* post hoc*).

As expected, weight, BMI, WC, and WHtR were higher in overweight and obesity groups (Figure 1 and Table 2). BMI was positively correlated with WC (*r* = 0.835, *p* = 0.000) and WHtR (*r* = 0.8640, *p* = 0.000) in the whole sample and also in all groups stratified by sex or age (data not shown). The strongest correlation was found for WHtR in women between 41 and 64 years of age (*r* = 0.875, *p* = 0.000).

The prevalence of abdominal obesity depends on the criteria applied: 28.1% of the population had high WC and 58.4% had WHtR ≥ 0.5. The prevalence was higher in women (especially those in the older female group) when only the WC criterion is considered; contrarily, the prevalence was higher among men when applying the WHtR criterion (Table 1).

**Figure 2:**
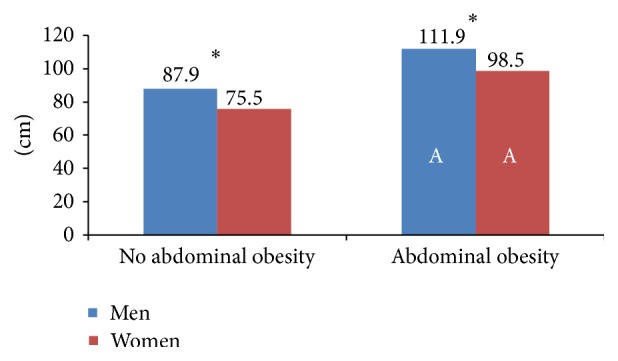
Waist circumference (WC) in Spanish adults according to WC classification. ^*∗*^Significant differences between men and women in the same WC group (Mann-Whitney *U* test). A: significant differences in the same sex group between adults with or without abdominal obesity (>88 cm for women and >102 cm for men) (Mann-Whitney *U* test).

**Figure 3:**
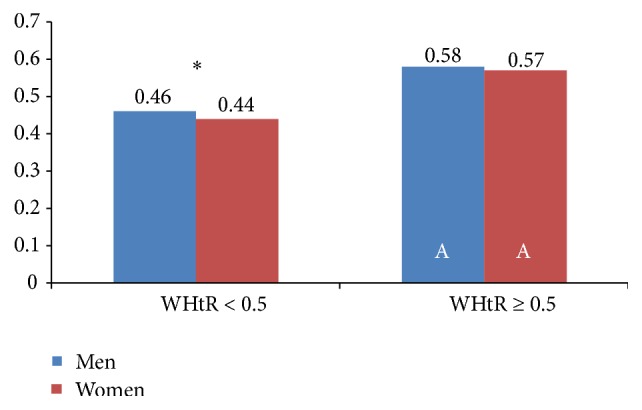
Waist/height ratio (WHtR) in Spanish adults according to WHtR classification. ^*∗*^Significant differences between men and women in the same WHtR group (Mann-Whitney *U* test). A: significant differences in the same sex group between adults with WHtR < 0.5 and WHtR ≥ 0.5 (Mann-Whitney *U* test).

Weight, BMI, WC, and WHtR were higher in those with abdominal obesity, regardless of the criteria used (Tables 3 and 4 and Figures 2 and 3). It is noteworthy that 42.1% (53.2% of men and 30.7% of women) of participants with low WC had a high WHtR (≥0.5) and some participants were also overweight (28.6%) or obese (3.2%) (Table 3).

The likelihood of having a high WC was lower in men (OR 0.712, 0.573–0.884; *p* = 0.002) and increased with age (OR 1.060, 1.050–1.071; *p* = 0.000) and for both sexes (OR 1.067, 1.052–1.081; *p* = 0.000 for women and OR = 1.053, 1.038–1.068; *p* = 0.000 for men).

Conversely, the likelihood of having a high WHtR was higher in men (OR 4.589, 3.715–5.670; *p* = 0.000) and increased with age (OR 1.062, 1.052–1.072; *p* = 0.000). The highest risk was found in men 41–64 years old (OR 7.606, 5.081–11.386; *p* = 0.000).

Regarding the composite index, only 36.1% of participants (28.9% of men and 42.7% of women) had an optimal anthropometric situation (WHtR < 0.5 and BMI < 25 kg/m^2^; level 1), whereas 50.1% had both excess weight (BMI ≥ 25 kg/m^2^) and high WHtR (levels 4 and 5) (Table 1). The remainder of the sample (13.9%) had intermediate situations: high BMI and no abdominal obesity (level 2) or normal BMI and abdominal obesity with metabolic risk (level 3) (Table 1).

This optimal situation (level 1) decreased with age; 49.3% of those in the 18–40-year-old group were in level 1 (56.9% of women and 41.4% of men) versus 21% in the 41–64-year-old group (27.1% of women, 14.0% of men) (Table 1). In contrast, 36.3% of those in the youngest group presented the most unfavorable situation (levels 4 and 5) versus 65.9% of those in the oldest group, and the results were consistently worse for men (Table 1).

Men had a higher risk of being classified as level 5 (OR 1.953, 1.486–2.568; *p* = 0.000) or levels 4 and 5 (OR 1.909, 1.541–2.365; *p* = 0.000). The risk of being at level 5 increased with each year of age (OR 1.096; 1.081–1.110; *p* = 0.000); this was also true for being classified as levels 4 and 5 (OR = 1.084, 1.072–1.095; *p* = 0.000).

Table 5 presents data regarding the composite index. Abdominal obesity when considering WC was found among 10.2%, 32.4%, and 88.3% of participants in levels 3, 4, and 5, respectively; contrarily, when using WHtR, 100% of participants of those levels were classified as being at risk.

## 4. Discussion

The present study provides objective data on measures of weight, height, and waist circumference in a representative sample of the adult Spanish population and provides updated information on the prevalence of overweight, obesity, and abdominal adiposity among Spanish adults.

More than half of Spanish adults (between 18 and 64 years old) in our population had weight excess (BMI > 25 kg/m^2^) (Table 1). The increased prevalence of overweight and obesity in the Spanish population is particularly worrisome, especially when comparing our results (35.8% of overweight people and 19.9% obese) and those of ENRICA study (Study on Nutrition and Cardiovascular Risk in Spain) (carried out in 2008–2010) (37.6% and 19.7%) [21] with those found in 2009 in a Spanish representative sample [4, 5] (34.2% and 13.6%).

The prevalence of overweight and obesity in our study is higher among men. In developing countries, the prevalence of obesity is usually higher in the female population, whereas the trend in developed countries varies [32]. For example, in Portugal [33], the Netherlands [34], and Bulgaria [35], the prevalence of overweight and obesity is higher among males, as in our study, while in Poland [2] it is higher among females. However, the results of other studies conducted in Spain show a variable trend: the 2012 ENRICA study showed that obesity affects up to 23,1% of men and 16.3% of women between 18 and 64 years of age [21]; the DORICA (Dyslipidemia, Obesity and Cardiovascular Risk in Spain) study noted that the prevalence of obesity was higher in women than men (17.5% versus 13.2%) [36]; and a study by Rodríguez-Rodríguez et al. [5] found no differences in the prevalence of obesity according to sex, although the prevalence of overweight was higher among men.

BMI is widely used to estimate the prevalence of obesity within a population and the risks associated with it; however, BMI does not account for the wide variation in body fat distribution [6]. Obese individuals with excess fat in the intra-abdominal depots are at particular risk of the adverse health consequences of obesity [12, 29, 31, 37]. WC [6] and WHtR are commonly used as indicators of abdominal adiposity [38].

In our study, the prevalence of abdominal obesity differed greatly depending on the criteria used, with 28% when WC was considered, and much higher (58.4%) using WHtR. These figures are slightly higher than those recently found among Portuguese population (42.1% of adults with high WHtR) [33] but similar to those found in other Spanish groups, which also observed the same discrepancy between these two indicators [4]. For example, Rodríguez-Rodríguez et al. [5] found 22.2% abdominal obesity considering WC as a reference and 54.7% using WHtR ≥ 0.5. And more recently the ENRICA study [21] found that 28.8% of adults aged 18–64 years had abdominal obesity considering WC. This highlights a worrying trend among Spanish adults of increased prevalence not only of overweight and obesity but also of abdominal adiposity. There is thus an urgent need to implement measures to slow or reverse this trend.

In addition, we found that the prevalence of abdominal obesity when WC and WHtR were applied varied by sex. In our study, the prevalence of abdominal obesity was higher among men when WHtR was considered (64.7% versus 52.4% in women; *p* < 0.001); in contrast, this was higher in women when a sex-specific cutoff point for WC was applied (31.4% versus 24.6% in men; *p* < 0.001). We must bear in mind that populations differ in their levels of risk associated with a particular WC, such that it is impossible to develop globally applicable cutoff points [6]. It could be that the cutoffs used to define abdominal obesity in the Spanish population do not properly describe the actual situation. In fact, other authors have proposed different cutoffs, to better identify abdominal obesity in other populations [39]. Although WC provides a simple and practical method of identifying those people at increased risk of obesity-associated illness [40], some studies have demonstrated that WHtR is a better predictor of disease or mortality risk than WC or BMI [28, 41, 42]. WHtR may also be a more useful global clinical screening tool than WC because it corrects by height and prevents underestimation of the risk in individuals who are taller or shorter than average [30]. Moreover, WHtR is potentially advantageous as it may not require conversion to sex- or population-specific cutoffs or percentiles [43]. Several studies have indicated that the risks described above are increased when the WHtR is equal to or greater than 0.5 in both men and women [29–31].

Here, the prevalence of global and abdominal obesity was higher among older participants. This has been widely confirmed by other authors [2, 4, 5, 33, 39, 44–46], who have generally found that both BMI and other abdominal obesity indicators increase from 20 to 29 years of age, reaching a peak at age 50–60 years [3, 5].

Different studies have confirmed that BMI and WC are independent predictors of adiposity in both adults [43, 47] and children [48]. But it has also been suggested that measurement of both BMI and WHtR in a composite index may be a better predictor of cardiovascular risk than BMI or WHtR alone. For example, Millar et al. 2015 [43] defined a composite index using both BMI and WHtR tertiles and suggested that the use of both measures combined improved discrimination of individual cardiometabolic risk factors and identified a subset of at-risk individuals who might otherwise be missed. In our study we have defined a composite index using well-defined cutoff points established for both BMI (WHO cutoff points of overweight and obesity) and WHtR (< or ≥0.5). This composite index allows us to describe the distribution of population in different levels of aggregation of factors (normal weight, overweight, or obese with or without abdominal adiposity), where level 1 represents the best anthropometric situation and level 5 the worst. It is likely that certain levels were associated with increased cardiometabolic risk, but unfortunately we cannot check it in the present study, since there is no information about the cardiometabolic risk factors of the studied population in ANIBES study.

In our study, 5.6% of participants (6.4% of men and 4.8% of women) were overweight or obese without abdominal adiposity (level 2 of our composite index) (Table 1). This figure is lower than that of Kowalkowska et al. [33], who found 16.4% of Portuguese adults in this situation. Those adults could have early general overweight but not yet have abdominal obesity; in fact, in our study, participants in level 2 were younger than those in levels 4 and 5 (Table 5). However, the adults in the previous study could be physically more active and have greater fat-free mass and thus weigh more. A total 8.3% of Spanish adults are normal weight with abdominal obesity (level 3 of our composite index), a figure similar to that found in a Portuguese population [33]. This latter group was more likely to suffer from major metabolic problems, such as ischemic heart disease, myocardial infarction, hypertension, dyslipidemia, and diabetes mellitus; however, their BMIs did not identify them as being at risk of these diseases [28, 40–42].

The research data in our study were collected in 2013 among a representative sample, providing new epidemiological data for Spanish adults. One of the strengths of the study is the careful design, protocol, and methodology used, conducted among a random representative sample of the Spanish population. In addition, height, weight, and WC were measured and were not self-reported, which is a more accurate assessment procedure that also strengthens these data. And finally we use both WC and WHtR to assess abdominal adiposity. However, our study has some limitations, as its cross-sectional design, which provides evidence for association but not causal relationships. Residual and confounding by unobserved and unmeasured factors is also likely. Finally, there are no biochemical data whether blood pressure was measured, so we cannot analyze the association between the anthropometric parameters or our composite index and the cardiometabolic risk indicators of the study population.

## 5. Conclusions

The present study confirms the high prevalence of overweight and obesity among the Spanish population. Furthermore, the situation is worse than even a few years ago, so it is necessary to implement more effective strategies for preventing and reducing high adiposity levels and the corresponding health consequences at a national level. In this regard, it is important to identify those factors that contribute to the problem, so as to design appropriate strategies. The results of this study will allow us to have an understanding of the current anthropometric situation of the Spanish population, as a first step in planning interventions and assessing their effectiveness in the future.

## Figures and Tables

**Table 1:** Anthropometric data of Spanish adults.

	Adults total (18–64 y)		Adults 1 (18–40 y)		Adults 2 (41–64 y)	
	Men	Women	Men	Women	Men	Women
*n* (%)	798 (48.2)	857 (51.8)	435 (26.3)	448 (27.1)	363 (21.9)	409 (24.7)
Age (years)^(1)^	39.6 ± 12.2	40.3 ± 12.2	30.2 ± 6.7	30.4 ± 6.4	50.9 ± 6.1	51.1 ± 6.5
Median (P25–P75)	39 (30–50)	40 (31–50)	31 (25–36)	31 (25–36)	50 (46–56)	51 (45–56)
Weight (kg)^(1)^	82.4 ± 15.34	66.6 ± 13.62^S^	80.4 ± 14.81	63.9 ± 12.96^S^	84.7 ± 15.65^E^	69.6 ± 13.73^SE^
Median (P25–P75)	80.3 (72–90.3)	64.4 (56.8–73.6)	78.3 (70.7–87)	61.7 (54.8–70)	83 (74.1–93.2)	67.5 (59.6–76.9)
Height (cm)^(1)^	174.5 ± 6.95	161.3 ± 6.37^S^	175.8 ± 6.91	162.4 ± 6.44^S^	173 ± 6.69^E^	160.2 ± 6.09^SE^
Median (P25–P75)	175 (170–180)	161 (157–166)	176 (171–180)	163 (158–167)	173 (168–178)	160 (156–164)

BMI (kg/m^2^)^(1)^	27.1 ± 4.87	25.6 ± 5.3^S^	26 ± 4.62	24.2 ± 4.71^S^	28.3 ± 4.88^E^	27.2 ± 5.47^SE^
Median (P25–P75)	26.4 (23.7–29.5)	24.7 (21.8–28.1)	25.1 (22.8–28)	23.3 (20.8–26.5)	27.8 (25–30.8)	26.1 (23.2–30)
Normal weight (%)	36.4	48.3^S^	46.7	58.2^S^	24.2^E^	37.3^SE^
Overweight (%)	40.4	31.5^S^	37.4	25.8^S^	44	37.7^E^
Obesity (%)	22.7	17.2^S^	15.2	10.4^S^	31.6^E^	24.8^SE^

Waist circumference (cm)^(1)^	93.8 ± 13.61	82.7 ± 13.19^S^	89.4 ± 12.56	78.2 ± 11.93^S^	99.1 ± 12.94^E^	87.7 ± 12.76^SE^
Median (P25–P75)	93 (84–101.6)	80.5 (72.7–90.4)	87.3 (80.5–96.7)	76.2 (69.9–84.1)	97.4 (90.5–106)	86.6 (78–96.3)
Abdominal obesity (%)^(2)^	24.6	31.4^S^	15.7	18.7	35.2^E^	45.3^SE^

Waist/height (WHtR)^(1)^	0.54 ± 0.08	0.51 ± 0.09^S^	0.51 ± 0.07	0.48 ± 0.08^S^	0.57 ± 0.08^E^	0.55 ± 0.08^SE^
Median (P25–P75)	0.53 (0.48–0.59)	0.50 (0.45–0.56)	0.5 (0.46–0.55)	0.47 (0.43–0.52)	0.56 (0.52–0.62)	0.53 (0.49–0.61)
WHtR ≥ 0.5	64.7	52.4^S^	49.2	36.4^S^	83.2^E^	70.1^SE^

Composite index:						
Level 1 (%)	28.9	42.7^S^	41.4	56.9^S^	14.0^E^	27.1^SE^
Level 2 (%)	6.4	4.8	9.4	6.7	2.8^E^	2.7^E^
Level 3 (%)	7.9	8.6	6	6.9	10.2^E^	10.5
Level 4 (%)	34.2	26.8^S^	27.8	19.6^S^	41.9^E^	34.7^SE^
Level 5 (%)	22.6	17.0^S^	15.4	9.8^S^	31.1^E^	24.9^E^

(1) Mean ± SD; BMI: body mass index; (2) abdominal obesity: >88 cm for women and >102 cm for men; composite index: Level 1: WHtR < 0.5 and BMI < 25 kg/m^2^, Level 2: WHtR < 0.5 and BMI ≥ 25 kg/m^2^, Level 3: WHtR ≥ 0.5 and BMI < 25 kg/m^2^, Level 4: WHtR ≥ 0.5 and BMI ≥ 25 kg/m^2^ and BMI < 30 kg/m^2^, and Level 5: WHtR ≥ 0.5 and BMI ≥ 30 kg/m^2^.

Significant differences regarding sex (S) and age groups (E) (Mann-Whitney *U* test and 2-sample *Z*-test).

**Table 2:** Anthropometric data of Spanish men and women according to BMI classification.

	Men			Women		
	Normal weight	Overweight	Obesity	Normal weight	Overweight	Obesity
*n*	290	323	181	414	269	148
Age (years)^(1)^	34.5 ± 11.9	41.4 ± 11.1^A^	45.0 ± 11.4^AB^	37.1 ± 11.6^S^	42.8 ± 11.9^A^	46.5 ± 11.0^AB^
Median (P25–P75)	33.0 (24.0–43.0)	40.0 (33.0–50.0)	46.0 (37.0–55.0)	36.0 (28.0–45.0)	44.0 (33.0–53.0)	47.5 (37.3–55.8)
Weight (kg)^(1)^	70.0 ± 7.04	82.9 ± 7.32^A^	101.8 ± 14.9^AB^	57.8 ± 6.01^S^	70.2 ± 6.27^AS^	88 ± 11.73^ABS^
Median (P25–P75)	70.5 (65.7–74.6)	82.9 (77.9–87.2)	100 (92.3–107.5)	57.7 (53.5–61.9)	70 (65–74)	86.8 (79.1–93.8)
Height (cm)^(1)^	175.5 ± 6.6	174.5 ± 7.0	172.9 ± 7.2^AB^	162.1 ± 5.9^S^	160.9 ± 6.6^AS^	159.5 ± 6.6^ABS^
Median (P25–P75)	175 (171–180)	175 (169–180)	173 (168–178)	162 (158–167)	161 (156–166)	160 (155–164)

Waist circumference (cm)^(1)^	82.6 ± 7.1	94.6 ± 7.1^A^	111 ± 11.8^AB^	74.1 ± 6.7^S^	87 ± 7.6^AS^	101.9 ± 10.8^ABS^
Median (P25–P75)	82.2 (77.6–87.2)	95.0 (90.0–99.1)	109.7 (103.3–117)	73.5 (69.3–78.9)	87.0 (81.3–91.4)	101.3 (94.7–107.8)
Abdominal obesity (%)	0.3	14.3^A^	82.3^AB^	2.9^S^	43.7^AS^	93.9^AB^

Waist/height (WHtR)^(1)^	0.47 ± 0.04	0.54 ± 0.04^A^	0.64 ± 0.07^AB^	0.46 ± 0.04^S^	0.54 ± 0.05^A^	0.64 ± 0.07^AB^
Median (P25–P75)	0.47 (0.44–0.49)	0.54 (0.52–0.57)	0.64 (0.60–0.68)	0.46 (0.43–0.49)	0.53 (0.51–0.57)	0.63 (0.59–0.68)
WHtR ≥ 0.5	22.1	84.5^A^	99.4^AB^	17.6	85.6^A^	98.6^AB^

Composite index:						
Level 1 (%)	78.3	0^A^	0^A^	82.1	0^A^	0^A^
Level 2 (%)	0	15.5^A^	0.6^B^	0	14.5^A^	1.4^B^
Level 3 (%)	21.7	0^A^	0^A^	17.9	0^A^	0^A^
Level 4 (%)	0	84.5^A^	0^B^	0	85.5^A^	0^B^
Level 5 (%)	0	0	99.4^AB^	0	0	98.6^AB^

(1) Mean ± SD; BMI: body mass index; abdominal obesity: >88 cm for women and >102 cm for men; composite index: Level 1: WHtR <0.5 and BMI < 25 kg/m^2^, Level 2: WHtR < 0.5 and BMI ≥ 25 kg/m^2^, Level 3: WHtR ≥ 0.5 and BMI < 25 kg/m^2^, Level 4: WHtR ≥ 0.5 and BMI ≥ 25 kg/m^2^ and BMI < 30 kg/m^2^, and Level 5: WHtR ≥ 0.5 and BMI ≥ 30 kg/m^2^.

Significant differences in the same sex group: A: regarding normal weight, B: regarding overweight, (Kruskal-Wallis test, and Dunn-Bonferroni *post hoc*). Significance between men and women in the same BMI group (S) (Mann-Whitney *U* test and 2-sample *Z*-test).

**Table 3:** Anthropometric data of Spanish adults (total population) according to waist circumference classification.

	Men		Women	
	No abdominal obesity	Abdominal obesity^(2)^	No abdominal obesity	Abdominal obesity^(2)^
*n*	602	196	588	269
Age (years)^(1)^	37.86 ± 12.06	45.11 ± 10.91^*∗*^	37.51 ± 11.65	43.31 ± 11.18^*∗*^
Median (P25–P75)	37.0 (28.0–47.3)	46.0 (37.0–55.0)	37.0 (29.0–46.0)	47.0 (38.0–56.0)
Weight (kg)^(1)^	76.5 ± 10.0	100.4 ± 15.2^*∗*^	60.3 ± 8.5^S^	80.3 ± 12.7^*∗*S^
Median (P25–P75)	76.5 (70.0–83.1)	99.1 (91.3–106.8)	59.8 (54.3–65.7)	78.8 (71.0–87.5)
Height (cm)^(1)^	174.6 ± 6.87	174.3 ± 7.22	161.5 ± 6.16^S^	161 ± 6.81^S^
Median (P25–P75)	175.0 (170.0–180.0)	174.0 (170.0–180.0)	162.0 (157.0–166.0)	161.0 (156.0–166.0)

BMI (kg/m^2^)^(1)^	25.1 ± 3.01	33 ± 4.63^*∗*^	23.1 ± 3.11^S^	31.1 ± 5^*∗*S^
Median (P25–P75)	25.0 (23.0–27.2)	32.5 (30.0–35.3)	22.9 (20.8–25.2)	30.1 (27.6–33.6)

Waist/height (WHtR)^(1)^	0.50 ± 0.05	0.64 ± 0.06^*∗*^	0.47 ± 0.05^S^	0.61 ± 0.07^*∗*S^
Median (P25–P75)	0.51 (0.47–0.54)	0.63 (0.60–0.67)	0.47 (0.43–0.51)	0.60 (0.57–0.64)
WHtR ≥ 0.5 (%)	53.2	100.0^*∗*^	30.7^S^	100.0^*∗*^

Composite index:				
Level 1 (%)	38.4	0^*∗*^	62.2^S^	0^*∗*^
Level 2 (%)	8.5	0^*∗*^	7	0^*∗*^
Level 3 (%)	10.3	0.5^*∗*^	10.4	4.8^*∗*S^
Level 4 (%)	37.7	23.5^*∗*^	19.2^S^	43.5^*∗*S^
Level 5 (%)	5.1	76.0^*∗*^	1.2^S^	51.7^*∗*S^

(1) Mean ± SD; BMI: body mass index; (2) abdominal obesity: >88 cm for women and >102 cm for men; composite index: Level 1: WHtR < 0.5 and BMI < 25 kg/m^2^, Level 2: WHtR < 0.5 and BMI ≥ 25 kg/m^2^, Level 3: WHtR ≥ 0.5 and BMI < 25 kg/m^2^, Level 4: WHtR ≥ 0.5 and BMI ≥ 25 kg/m^2^ and BMI < 30 kg/m^2^, and Level 5: WHtR ≥ 0.5 and BMI ≥ 30 kg/m^2^.

Significant differences between adults with or without abdominal obesity. (*∗*) Significant differences between men and women in the waist circumference group (S) (Mann-Whitney *U* test and 2-sample *Z*-test).

**Table 4:** Anthropometric data of Spanish adults according to WHtR classification.

	Men		Women	
	WHtR < 0.5	WHtR ≥ 0.5	WHtR < 0.5	WHtR ≥ 0.5
*n*	282	516	407	450
Age (years)^(1)^	32.5 ± 11.2	43.5 ± 10.9	35.0 ± 10.7^S^	45.0 ± 11.53^S^
Median (P25–P75)	31.0 (23.0–40.0)	44.0 (36.0–52.0)	34.0 (25.0–42.0)	46.0 (36.0–55.0)
Weight (kg)^(1)^	71.5 ± 8.5	88.3 ± 15.1^*∗*^	58.5 ± 8.29^S^	73.9 ± 13.2^*∗*S^
Median (P25–P75)	71.3 (65.9–77.0)	86.3 (79.0–96.0)	57.9 (52.9–62.9)	71.5 (64.8–80.7)
Height (cm)^(1)^	176.1 ± 6.40	173.6 ± 7.10^*∗*^	163.0 ± 5.85^S^	159.85 ± 6.44^*∗*S^
Median (P25–P75)	176 (172–180)	173 (169–179)	163 (159–167)	160 (155–164)

BMI (kg/m^2^)^(1)^	23.0 ± 21.8	29.3 ± 4.53^*∗*^	22.0 ± 2.62^S^	28.9 ± 4.91^*∗*^
Median (P25–P75)	23.0 (21.7–24.5)	28.3 (26.3–31.2)	21.8 (20.3–23.4)	27.8 (25.7–31.5)

Waist circumference (cm)^(1)^	80.6 ± 5.6	101.0 ± 11.2^*∗*^	72.3 ± 5.6^S^	92.1 ± 10.7^*∗*S^
Median (P25–P75)	81.1 (77.2–84.5)	99 (93.3–106.2)	72.2 (68.1–76.4)	90.0 (84.0–98.0)
Abdominal obesity (%)^(2)^	0	38.0^*∗*^	0	59.8^*∗*S^

Composite index:				
Level 1 (%)	81.9	0^*∗*^	89.9^S^	0^*∗*^
Level 2 (%)	18.1	0^*∗*^	10.1^S^	0^*∗*^
Level 3 (%)	0	12.2^*∗*^	0	16.4^*∗*^
Level 4 (%)	0	52.9^*∗*^	0	51.1^*∗*^
Level 5 (%)	0	34.9^*∗*^	0	32.4^*∗*^

(1) Mean ± SD; BMI: body mass index; (2) abdominal obesity: >88 cm for women and >102 cm for men; composite index: Level 1: WHtR < 0.5 and BMI < 25 kg/m^2^, Level 2: WHtR < 0.5 and BMI ≥ 25 kg/m^2^, Level 3: WHtR ≥ 0.5 and BMI < 25 kg/m^2^, Level 4: WHtR ≥ 0.5 and BMI ≥ 25 kg/m^2^ and BMI < 30 kg/m^2^, and Level 5: WHtR ≥ 0.5 and BMI ≥ 30 kg/m^2^.

Significant differences between adults with WHtR < 0.5 and WHtR ≥ 0.5 (*∗*). Significant differences between men and women in the same WHtR group (S) (Mann-Whitney *U* test and 2-sample *Z*-test).

**Table 5:** Anthropometric data of Spanish men and women according to composite index classification.

	Composite index men					Composite index women				
	Level 1	Level 2	Level 3	Level 4	Level 5	Level 1	Level 2	Level 3	Level 4	Level 5
*n*	231	51	63	273	180	366	41	74	230	146
Age (years)^(1)^	32.2 ± 11.1	34.1 ± 11.2	42.9 ± 10.6^AB^	42.8 ± 10.6^AB^	44.9 ± 11.4^ABCD^	35.1 ± 10.7^S^	34.7 ± 11.0	44.1 ± 12.4^AB^	44.2 ± 11.5^AB^	46.7 ± 10.9^AB^
Median	31	34	43	44	46	34	33	43.5	45	48
(P25–P75)	(23–39)	(25–40)	(37–50)	(35–51)	(37–55)	(25.8–42)	(25–42.5)	(34–56.3)	(35–54)	(37.8–56)
Weight (kg)^(1)^	69.3 ± 7.4	81.6 ± 5.2^A^	71.2 ± 6.9^B^	83.2 ± 7.6^AC^	102 ± 14.8^ABCD^	56.8 ± 6.4^S^	73.5 ± 7.8^AS^	59.4 ± 6.0^ABS^	69.8 ± 6.3^ABCS^	83.7 ± 11.6^ABCDS^
Median	70	82.2	71.8	83	100.1	56.7	71.8	59.4	69.5	86.5
(P25–P75)	(65.0–74.0)	(77.4–84.6)	(67.7–75.4)	(78.0–87.7)	(92.3–107.5)	(52.3–61.0)	(69.6–76.4)	(55.7–64.0)	(65.0–74.2)	(79.1–93.7)
Height (cm)^(1)^	176.0 ± 6.6	176.5 ± 5.5	173.3 ± 6.8^AB^	174.1 ± 7.2^AB^	173.0 ± 7.1^AB^	162.8 ± 5.9^S^	164.8 ± 5.4^AS^	159.7 ± 5.9^ABS^	160.2 ± 6.6^ABS^	159.3 ± 6.5^ABS^
Median	176	175	173	174	173	163	164	160	160	160
(P25–P75)	(172–180)	(172–180)	(170–178)	(168–179)	(168.3–178)	(159–167)	(161–168)	(155.8–164.3)	(156–165)	(154.8–164)

BMI (kg/m^2^)^(1)^	22.3 ± 1.7	26.2 ± 1.1^A^	23.7 ± 1.2^AB^	27.4 ± 1.4^ABC^	34.0 ± 4.1^ABC^	21.4 ± 1.9^S^	27.1 ± 2.4^AS^	23.3 ± 1.3^AB^	27.2 ± 1.4^AC^	34.6 ± 4.2^ABCD^
Median	22.5	25.9	24.1	27.4	33.4	21.4	26.5	23.7	27.2	33.4
(P25–P75)	(21.4–23.7)	(25.4–26.9)	(23.2–24.5)	(26.3–28.5)	(31.0–35.6)	(20.2–23.0)	(25.6–27.9)	(22.3–24.4)	(26.0–28.2)	(31.6–36.5)

WC (cm)^(1)^	79.8 ± 5.5	84.3 ± 4.3^A^	91.7 ± 4.6^AB^	96.5 ± 5.7^ABC^	111.2 ± 11.5^ABC^	71.7 ± 5.5^S^	77.5 ± 3.8^AS^	83.6 ± 4.6^ABS^	88.6 ± 6.7^ABCS^	102.2 ± 10.6^ABCDS^
Median	80	85	91.3	96.1	109.9	71.6	78	83.3	88	101.4
(P25–P75)	(76.0–84.0)	(82.5–86.7)	(88.3–95.1)	(92.4–100.3)	(103.4–117)	(67.9–75.5)	(75.8–79.8)	(80.4–86.2)	(83.5–92.5)	(95.0–108.0)
Abdominal obesity (%)^(2)^	0	0	1.6	16.8^C^	82.8^CD^	0	0	17.6^S^	50.9^CS^	95.2^CDS^

WHtR^(1)^	0.45 ± 0.03	0.48 ± 0.02^A^	0.53 ± 0.02^AB^	0.55 ± 0.03^ABC^	0.64 ± 0.07^ABC^	0.44 ± 0.03^S^	0.47 ± 0.02^AS^	0.52 ± 0.02^ABS^	0.55 ± 0.04^ABC^	0.64 ± 0.07^ABCD^
Median	0.46	0.49	0.53	0.55	0.64	0.44	0.48	0.52	0.54	0.63
(P25–P75)	(0.43–0.48)	(0.47–0.49)	(0.51–0.54)	(0.53–0.58)	(0.60–0.68)	(0.42–0.47)	(0.46–0.48)	(0.51–0.53)	(0.52–0.58)	(0.59–0.68)

(1): Mean ± SD; BMI: body mass index; WC: waist circumference; (2) abdominal obesity: >88 cm for women and >102 cm for men; WHtR: Waist to height ratio; Composite index: Level 1: WHtR < 0.5 and BMI < 25 kg/m^2^, Level 2: WHtR < 0.5 and BMI ≥ 25 kg/m^2^, Level 3: WHtR ≥ 0.5 and BMI < 25 kg/m^2^, Level 4: WHtR ≥ 0.5 and BMI ≥ 25 kg/m^2^ and BMI < 30 kg/m^2^, and Level 5: WHtR ≥ 0.5 and BMI ≥ 30 kg/m^2^.

Significant differences in the same sex group: A: regarding Level 1, B: regarding Level 2, C: regarding Level 3, D: regarding Level 4 (Kruskal-Wallis test, and Dunn-Bonferroni *post hoc*). Significant differences between men and women in the same composite index level (S) (Mann-Whitney *U* test and 2-sample *Z*-test).

## References

[1] Finucane M. M., Stevens G. A., Cowan M. J. (2011). National, regional, and global trends in body-mass index since 1980: systematic analysis of health examination surveys and epidemiological studies with 960 country-years and 9·1 million participants. *The Lancet*.

[2] Krzysztoszek J., Wierzejska E., Zielińska A. (2015). Obesity. An analysis of epidemiological and prognostic research. *Archives of Medical Science*.

[3] Ministry of Health (2013). *Encuesta Nacional de Salud 2011-2012*.

[4] Ortega Anta R. M., López-Sobaler A. M., Pérez-Farinós N. (2013). Associated factors of obesity in Spanish representative samples. *Nutricion Hospitalaria*.

[5] Rodríguez-Rodríguez E., López-Plaza B., López-Sobaler A. M., Ortega R. M. (2011). Overweight and obesity among Spanish adults. *Nutricion Hospitalaria*.

[6] World Health Organization (2000). Obesity: preventing and managing the global epidemic: report of a WHO consultation. *WHO Technical Report Series*.

[7] Anderson W. L., Wiener J. M., Khatutsky G., Armour B. S. (2013). Obesity and people with disabilities: the implications for health care expenditures. *Obesity*.

[8] Anderson A. S., Key T. J., Norat T. (2015). European code against cancer 4th edition: obesity, body fatness and cancer. *Cancer Epidemiology*.

[9] Guh D. P., Zhang W., Bansback N., Amarsi Z., Birmingham C. L., Anis A. H. (2009). The incidence of co-morbidities related to obesity and overweight: a systematic review and meta-analysis. *BMC Public Health*.

[10] Koyanagi A., Moneta M. V., Garin N. (2015). The association between obesity and severe disability among adults aged 50 or over in nine high-income, middle-income and low-income countries: a cross-sectional study. *BMJ Open*.

[11] Yatsuya H., Li Y., Hilawe E. H. (2014). Global trend in overweight and obesity and its association with cardiovascular disease incidence. *Circulation Journal*.

[12] Papaetis G. S., Papakyriakou P., Panagiotou T. N. (2015). Central obesity, type 2 diabetes and insulin: exploring a pathway full of thorns. *Archives of Medical Science*.

[13] Greenberg J. A. (2013). Obesity and early mortality in the United States. *Obesity*.

[14] Taylor V. H., Forhan M., Vigod S. N., McIntyre R. S., Morrison K. M. (2013). The impact of obesity on quality of life. *Best Practice and Research: Clinical Endocrinology and Metabolism*.

[15] Chapman C. D., Benedict C., Brooks S. J., Schiöth H. B. (2012). Lifestyle determinants of the drive to eat: a meta-analysis. *American Journal of Clinical Nutrition*.

[16] Wang Y. C., McPherson K., Marsh T., Gortmaker S. L., Brown M. (2011). Health and economic burden of the projected obesity trends in the USA and the UK. *The Lancet*.

[17] González-Gross M., Castillo M. J., Moreno L. (2003). Feeding and assessment of nutritional status of spanish adolescents (AVENA study). Evaluation of risks and interventional proposal. I.Methodology. *Nutrición Hospitalaria. Suplementos*.

[18] Pérez-Farinós N., López-Sobaler A. M., Dal Re M. Á. (2013). The ALADINO study: a national study of prevalence of overweight and obesity in spanish children in 2011. *BioMed Research International*.

[19] Aranceta-Bartrina J., Serra-Majem L., Foz-Sala M. (2005). Prevalence of obesity in Spain. *Medicina Clinica*.

[20] Rodríguez-Rodríguez E., Ortega R. M., Palmeros C., López-Sobaler A. M. (2011). Risk factors of overweight and obesity in Spanish population. *Nutrición Clínica y Dietética Hospitalaria*.

[21] Gutiérrez-Fisac J. L., Guallar-Castillón P., León-Muñoz L. M., Graciani A., Banegas J. R., Rodríguez-Artalejo F. (2012). Prevalence of general and abdominal obesity in the adult population of Spain, 2008-2010: the ENRICA study. *Obesity Reviews*.

[22] Ruiz E., Ávila J. M., Castillo A. (2015). The ANIBES study on energy balance in Spain: design, protocol and methodology. *Nutrients*.

[23] Ruiz E., Ávila J. M., Valero T. (2015). Energy intake, profile, and dietary sources in the spanish population: findings of the ANIBES study. *Nutrients*.

[24] Varela-Moreiras G., Ávila J. M., Ruiz E. (2015). Energy balance, a new paradigm and methodological issues: the ANIBES study in Spain. *Nutricion Hospitalaria*.

[25] Biró G., Hulshof K. F. A. M., Ovesen L., Amorim Cruz J. A. (2002). Selection of methodology to assess food intake. *European Journal of Clinical Nutrition*.

[26] Marfell-Jones M., Olds T., Stewart A., Carter L. (2006). *International Standards for Anthropometric Assessment*.

[27] Keller K., Rodríguez López S., Carmenate Moreno M. M. (2015). Association between meal intake behaviour and abdominal obesity in Spanish adults. *Appetite*.

[28] Schneider H. J., Friedrich N., Klotsche J. (2010). The predictive value of different measures of obesity for incident cardiovascular events and mortality. *Journal of Clinical Endocrinology and Metabolism*.

[29] Ashwell M., Hsieh S. D. (2005). Six reasons why the waist-to-height ratio is a rapid and effective global indicator for health risks of obesity and how its use could simplify the international public health message on obesity. *International Journal of Food Sciences and Nutrition*.

[30] Browning L. M., Hsieh S. D., Ashwell M. (2010). A systematic review of waist-to-height ratio as a screening tool for the prediction of cardiovascular disease and diabetes: 0.5 could be a suitable global boundary value. *Nutrition Research Reviews*.

[31] Srinivasan S. R., Wang R., Chen W., Wei C. Y., Xu J., Berenson G. S. (2009). Utility of waist-to-height ratio in detecting central obesity and related adverse cardiovascular risk profile among normal weight younger adults (from the Bogalusa Heart Study). *American Journal of Cardiology*.

[32] Low S., Chin M. C., Deurenberg-Yap M. (2009). Review on epidemic of obesity. *Annals of the Academy of Medicine Singapore*.

[33] Kowalkowska J., Poínhos R., Franchini B. (2016). General and abdominal adiposity in a representative sample of Portuguese adults: dependency of measures and socio-demographic factors’ influence. *British Journal of Nutrition*.

[34] Schokker D. F., Visscher T. L. S., Nooyens A. C. J., van Baak M. A., Seidell J. C. (2007). Prevalence of overweight and obesity in the Netherlands. *Obesity Reviews*.

[35] Ivanova L., Dimitrov P., Dellava J., Hoffman D. (2008). Prevalence of obesity and overweight among urban adults in Bulgaria. *Public Health Nutrition*.

[36] Aranceta J., Pérez-Rodrigo C., Serra-Majem L., Aranceta J., Foz M., Gil B. (2004). Estudio DORICA: dislipemia, obesidad y riesgo cardiovascular. *Obesidad y Riesgo Cardiovascular*.

[37] Stępień A., Stępień M., Wlazeł R. N., Paradowski M., Banach M., Rysz J. (2014). Assessment of the relationship between lipid parameters and obesity indices in non-diabetic obese patients: a preliminary report. *Medical Science Monitor*.

[38] Gibson R. S. (2005). *Principles of Nutritional Assessment*.

[39] do Carmo I., Dos Santos O., Camolas J. (2008). Overweight and obesity in Portugal: national prevalence in 2003–2005. *Obesity Reviews*.

[40] Ness-Abramof R., Apovian C. M. (2008). Waist circumference measurement in clinical practice. *Nutrition in Clinical Practice*.

[41] Ashwell M., Gunn P., Gibson S. (2012). Waist-to-height ratio is a better screening tool than waist circumference and BMI for adult cardiometabolic risk factors: systematic review and meta-analysis. *Obesity Reviews*.

[42] Martínez-González M. A., García-Arellano A., Toledo E. (2014). Obesity indexes and total mortality among elderly subjects at high cardiovascular risk: The PREDIMED Study. *PLoS ONE*.

[43] Millar S. R., Perry I. J., Phillips C. M. (2015). Assessing cardiometabolic risk in middle-aged adults using body mass index and waist-height ratio: are two indices better than one? A cross-sectional study. *Diabetology and Metabolic Syndrome*.

[44] Wang H., Wang J., Liu M.-M. (2012). Epidemiology of general obesity, abdominal obesity and related risk factors in urban adults from 33 communities of northeast china: the CHPSNE study. *BMC Public Health*.

[45] Stevens J., Katz E. G., Huxley R. R. (2010). Associations between gender, age and waist circumference. *European Journal of Clinical Nutrition*.

[46] Martínez-Ros M. T., Tormo M. J., Navarro C., Chirlaque M. D., Pérez-Flores D. (2001). Extremely high prevalence of overweight and obesity in Murcia, a Mediterranean region in south-east Spain. *International Journal of Obesity*.

[47] Janssen I., Heymsfield S. B., Allison D. B., Kotler D. P., Ross R. (2002). Body mass index and waist circumference independently contribute to the prediction of nonabdominal, abdominal subcutaneous, and visceral fat. *The American Journal of Clinical Nutrition*.

[48] Aeberli I., Gut-Knabenhans M., Kusche-Ammann R. S., Molinari L., Zimmermann M. B. (2013). A composite score combining waist circumference and body mass index more accurately predicts body fat percentage in 6- to 13-year-old children. *European Journal of Nutrition*.

